# 2,4,6-Tri­nitro­phenyl 4-bromo­benzoate

**DOI:** 10.1107/S1600536813031061

**Published:** 2013-11-20

**Authors:** Rodolfo Moreno-Fuquen

**Affiliations:** a Departamento de Química – Facultad de Ciencias, Universidad del Valle, Apartado 25360, Santiago de Cali, Colombia

## Abstract

In the title benzoate derivative, C_13_H_6_BrN_3_O_8_, the benzene rings form a dihedral angle of 80.90 (9)°. The ester moiety forms dihedral angles of 3.2 (2) and 82.8 4(10)° with the benzene and picryl rings, respectively. The Br atom is disordered over two positions, with the site occupancy for the minor component being 0.48 (4). The crystal structure features C—H⋯O inter­actions, which generate a three-dimensional network.

## Related literature

For similar esters, see: Moreno-Fuquen *et al.* (2013 ▶). For hydrogen bonding, see: Nardelli (1995 ▶).
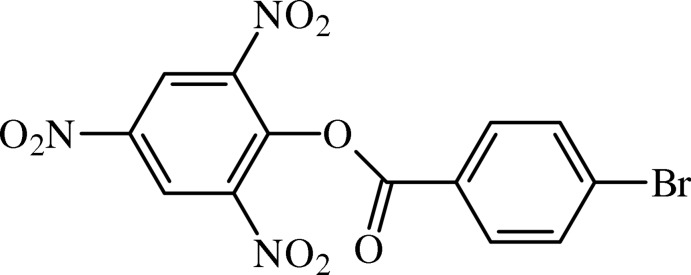


## Experimental

### 

#### Crystal data

C_13_H_6_BrN_3_O_8_
*M* *_r_* = 412.12
Monoclinic, 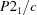

*a* = 10.8409 (5) Å
*b* = 10.0152 (7) Å
*c* = 13.9777 (7) Å
β = 99.246 (3)°
*V* = 1497.89 (15) Å^3^
*Z* = 4
Mo *K*α radiation
μ = 2.80 mm^−1^
*T* = 295 K
0.34 × 0.13 × 0.11 mm

#### Data collection

Nonius KappaCCD diffractometer
Absorption correction: multi-scan (*SADABS*; Bruker, 2004 ▶) *T*_min_ = 0.668, *T*_max_ = 0.753
10473 measured reflections
3340 independent reflections
2092 reflections with *I* > 2σ(*I*)
*R*_int_ = 0.060

#### Refinement

*R*[*F* ^2^ > 2σ(*F* ^2^)] = 0.046
*wR*(*F* ^2^) = 0.134
*S* = 1.02
3340 reflections
236 parameters
H-atom parameters constrained
Δρ_max_ = 0.30 e Å^−3^
Δρ_min_ = −0.50 e Å^−3^

### 

Data collection: *COLLECT* (Nonius, 2000 ▶); cell refinement: *SCALEPACK* (Otwinowski & Minor, 1997 ▶); data reduction: *DENZO* (Otwinowski & Minor, 1997 ▶) and *SCALEPACK*; program(s) used to solve structure: *SIR92* (Altomare *et al.*, 1994 ▶); program(s) used to refine structure: *SHELXL97* (Sheldrick, 2008 ▶); molecular graphics: *ORTEP-3 for Windows* (Farrugia, 2012 ▶) and *Mercury* (Macrae *et al.*, 2006 ▶); software used to prepare material for publication: *WinGX* (Farrugia, 2012 ▶).

## Supplementary Material

Crystal structure: contains datablock(s) I, global. DOI: 10.1107/S1600536813031061/tk5272sup1.cif

Structure factors: contains datablock(s) I. DOI: 10.1107/S1600536813031061/tk5272Isup2.hkl

Supplementary material file. DOI: 10.1107/S1600536813031061/tk5272Isup3.cml

Additional supplementary materials:  crystallographic information; 3D view; checkCIF report

## Figures and Tables

**Table 1:** Hydrogen-bond geometry (Å, °)

*D*—H⋯*A*	*D*—H	H⋯*A*	*D*⋯*A*	*D*—H⋯*A*
C10—H10⋯O2^i^	0.93	2.48	3.169 (4)	131
C12—H12⋯O1^ii^	0.93	2.49	3.273 (4)	142
C5—H5⋯O8^iii^	0.93	2.46	3.384 (4)	173
C3—H3⋯O6^iv^	0.93	2.42	3.291 (4)	157

Symmetry codes: (i) 
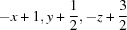
; (ii) 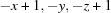; (iii) 
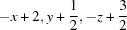
; (iv) 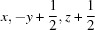.

**Fig. 1.**
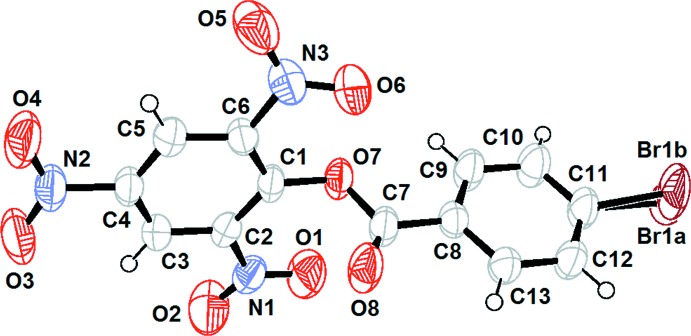
Molecular conformation and atom numbering scheme for the title compound with displacement ellipsoids drawn at the 50% probability level. H atoms are shown as spheres of arbitrary radius.

**Fig. 2.**
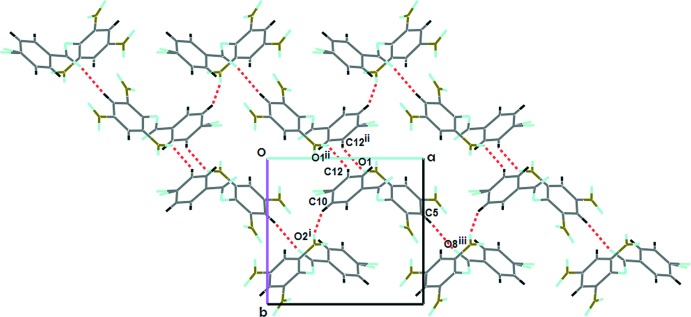
Part of the crystal structure of (I), showing the formation of helical chains which running along [100] and [010]. Symmetry codes: (i) -x+1, +y+1/2, -z+3/2; (ii) -x+1, -y, -z+1; (iii) -x+2, +y+1/2, -z+3/2.

**Fig. 3.**
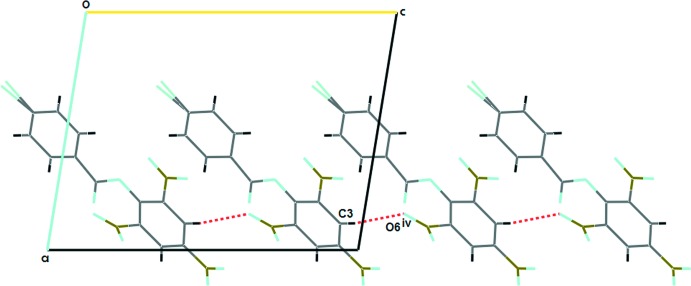
Part of the crystal structure of (I), showing the formation of chains of molecules which running along [001]. Symmetry code: (iv) x, -y+1/2,+z+1/2.

## supplementary crystallographic information

### 2.1. Synthesis and crystallization

The reagents and solvents for the synthesis were obtained from the Aldrich Chemical Co., and were used without additional purification. The title molecule was obtained through a two-step reaction. First 4-bromo­benzoic acid (0.223 g, 0.541 mmol) was refluxed in an excess of thio­nyl chloride (10 ml) for 1 h. Then, thio­nyl chloride was distilled off under reduced pressure to purify the 4-bromo­benzoyl chloride which was obtained as a pale-yellow translucent liquid. The same reaction flask was rearranged and a solution of picric acid (0.115 g, 0.541 mmol) in aceto­nitrile, was added drop-wise with constant stirring. The reaction mixture was left to reflux for about 1 h. A pale-yellow solid was obtained after leaving the solvent to evaporate. Crystals of good quality and suitable for single-crystal X-ray diffraction were grown from its aceto­nitrile solution. M.pt 457 (1) K.

### 2.2. Refinement

Crystal data, data collection and structure refinement details are summarized in Table 1. All H-atoms were positioned at geometrically idealized positions with C—H distances of 0.93 Å, and with *U*_iso_(H) = 1.2*U*_eq_(C). The Br atom was disordered over two positions [site occupancy for the minor component = 0.48 (4)].

### 3. Results and discussion

In the present work, the structure of the 2,4,6-tri­nitro­phenyl 4-bromo­benzoate (I) has been determined and is part of a series of picryl substituted-esters compounds that have been synthesized in our research group. The molecular structure of (I) is shown in Fig. 1. Bond lengths and angles are normal and are comparable with those reported for related compounds (Moreno-Fuquen *et al.*, 2013). The title molecule is twisted: the benzene rings form a dihedral angle of 80.90 (9)°. The central ester moiety C1—O7—C7(O8)—C8 is effectively planar (r.m.s. deviation of all non-hydrogen atoms = 0.0122 Å) and it forms angles of 3.2 (3) and 82.84 (10)° with the benzoate and the picryl rings, respectively. The nitro groups form dihedral angles with the attached benzene ring of 3.7 (3), 8.8 (3) and 20.8 (2)° for O1—N1—O2, O3—N2—O4 and O5—N3—O6, respectively.

In the crystal, in a first substructure, the molecules are inter­twined by weak C—H···O inter­actions, forming helical chains along [100]. The C10 atom of the benzoate ring acts as a hydrogen-bond donor to atom O2 at (-*x*+1, +*y*+1/2, -*z*+3/2). Growth in this direction is reinforced by the weak C5—H5···O8 inter­action, in which the C5 atom of the picryl ring acts as hydrogen-bond donor to carbonyl atom O8 at (*-x*+2,+*y*+1/2, -*z*+3/2). In this same substructure the C12 atom in the molecule acts as a hydrogen-bond donor to nitro-O1 atom at (-*x*+1, -*y*, -*z*+1), whose inter­action contributes to the growth of the crystal along [010]. The combination of these three contacts generate *R*^2^_2_(20) and *R*^6^_6_(38) rings along [100] (Fig. 2). In a second substructure shown in Fig. 3, it can be observed the formation of a chain of molecules through the weak C3—H3···O6 inter­actions. The C3 atom acts as hydrogen-bond donor to O6 atom of the nitro group in the molecule at (*x*, -*y*+1/2, +*z*+1/2), forming *C*(7) chains along [001]. Thus, the crystal system is a three-dimensional structure, sustained *via* C—H···O inter­actions (see Table 1; Nardelli, 1995). No Br···Br inter­actions are found in the crystal structure.

### Crystal data

**Table:**

C_13_H_6_BrN_3_O_8_	*F*(000) = 816
*M**_r_* = 412.12	*D*_x_ = 1.827 Mg m^−^^3^
Monoclinic, *P*2_1_/*c*	Melting point: 457(1) K
Hall symbol: -P 2ybc	Mo *K*α radiation, λ = 0.71073 Å
*a* = 10.8409 (5) Å	Cell parameters from 6103 reflections
*b* = 10.0152 (7) Å	θ = 2.9–27.5°
*c* = 13.9777 (7) Å	µ = 2.80 mm^−^^1^
β = 99.246 (3)°	*T* = 295 K
*V* = 1497.89 (15) Å^3^	Block, pale yellow
*Z* = 4	0.34 × 0.13 × 0.11 mm

### Data collection

**Table:**

Nonius KappaCCD diffractometer	3340 independent reflections
Radiation source: fine-focus sealed tube	2092 reflections with *I* > 2σ(*I*)
Graphite monochromator	*R*_int_ = 0.060
CCD rotation images, thick slices scans	θ_max_ = 27.6°, θ_min_ = 3.3°
Absorption correction: multi-scan (*SADABS*; Bruker, 2004)	*h* = −14→13
*T*_min_ = 0.668, *T*_max_ = 0.753	*k* = −11→13
10473 measured reflections	*l* = −18→16

### Refinement

**Table:**

Refinement on *F*^2^	Primary atom site location: structure-invariant direct methods
Least-squares matrix: full	Secondary atom site location: difference Fourier map
*R*[*F*^2^ > 2σ(*F*^2^)] = 0.046	Hydrogen site location: inferred from neighbouring sites
*wR*(*F*^2^) = 0.134	H-atom parameters constrained
*S* = 1.02	*w* = 1/[σ^2^(*F*_o_^2^) + (0.0632*P*)^2^ + 0.3604*P*] where *P* = (*F*_o_^2^ + 2*F*_c_^2^)/3
3340 reflections	(Δ/σ)_max_ < 0.001
236 parameters	Δρ_max_ = 0.30 e Å^−^^3^
0 restraints	Δρ_min_ = −0.50 e Å^−^^3^

### Special details

**Table:**

Geometry. All esds (except the esd in the dihedral angle between two l.s. planes) are estimated using the full covariance matrix. The cell esds are taken into account individually in the estimation of esds in distances, angles and torsion angles; correlations between esds in cell parameters are only used when they are defined by crystal symmetry. An approximate (isotropic) treatment of cell esds is used for estimating esds involving l.s. planes.
Refinement. Refinement of *F*^2^ against ALL reflections. The weighted *R*-factor *wR* and goodness of fit *S* are based on *F*^2^, conventional *R*-factors *R* are based on *F*, with *F* set to zero for negative *F*^2^. The threshold expression of *F*^2^ > σ(*F*^2^) is used only for calculating *R*-factors(gt) *etc*. and is not relevant to the choice of reflections for refinement. *R*-factors based on *F*^2^ are statistically about twice as large as those based on *F*, and *R*- factors based on ALL data will be even larger.

### Fractional atomic coordinates and isotropic or equivalent isotropic displacement parameters (Å^2^)

**Table:**

	*x*	*y*	*z*	*U*_iso_*/*U*_eq_	Occ. (<1)
Br1A	0.3092 (13)	0.2505 (17)	0.2655 (8)	0.100 (2)	0.48 (4)
Br1B	0.2896 (6)	0.2267 (6)	0.2775 (5)	0.0781 (10)	0.52 (4)
O7	0.70356 (18)	0.2879 (2)	0.69502 (15)	0.0547 (5)	
O8	0.8026 (2)	0.1287 (3)	0.62476 (17)	0.0831 (8)	
N1	0.6967 (3)	0.0905 (3)	0.8454 (2)	0.0606 (7)	
C13	0.6090 (3)	0.1222 (3)	0.4631 (2)	0.0517 (7)	
H13	0.6728	0.0598	0.4646	0.062*	
N3	0.9026 (2)	0.4791 (3)	0.7109 (2)	0.0630 (7)	
O6	0.8478 (3)	0.4642 (3)	0.62908 (16)	0.0870 (8)	
C6	0.8938 (3)	0.3763 (3)	0.78448 (19)	0.0475 (6)	
O1	0.6128 (3)	0.0871 (3)	0.7775 (2)	0.0940 (9)	
C8	0.6080 (3)	0.2073 (3)	0.5414 (2)	0.0478 (7)	
C5	0.9857 (3)	0.3788 (3)	0.8661 (2)	0.0515 (7)	
H5	1.0486	0.4429	0.8731	0.062*	
O2	0.7002 (3)	0.0195 (3)	0.9141 (2)	0.1052 (11)	
O4	1.1481 (3)	0.3782 (3)	1.0350 (2)	0.0979 (10)	
O5	0.9625 (3)	0.5792 (3)	0.7381 (2)	0.1012 (10)	
C12	0.5168 (3)	0.1292 (3)	0.3836 (2)	0.0579 (8)	
H12	0.5183	0.0729	0.3308	0.070*	
C7	0.7141 (3)	0.1995 (3)	0.6211 (2)	0.0525 (7)	
C3	0.8886 (3)	0.1888 (3)	0.9281 (2)	0.0496 (7)	
H3	0.8878	0.1250	0.9764	0.060*	
C4	0.9808 (3)	0.2838 (3)	0.9359 (2)	0.0501 (7)	
N2	1.0775 (3)	0.2846 (3)	1.0231 (2)	0.0663 (8)	
C1	0.7978 (2)	0.2826 (3)	0.77309 (18)	0.0456 (6)	
O3	1.0787 (3)	0.1913 (3)	1.0790 (2)	0.1036 (10)	
C2	0.7973 (3)	0.1898 (3)	0.84741 (19)	0.0472 (6)	
C9	0.5104 (3)	0.2978 (4)	0.5407 (2)	0.0615 (8)	
H9	0.5080	0.3537	0.5935	0.074*	
C10	0.4172 (3)	0.3040 (4)	0.4609 (3)	0.0714 (10)	
H10	0.3515	0.3640	0.4597	0.086*	
C11	0.4220 (3)	0.2211 (3)	0.3833 (2)	0.0621 (8)	

### Atomic displacement parameters (Å^2^)

**Table:**

	*U*^11^	*U*^22^	*U*^33^	*U*^12^	*U*^13^	*U*^23^
Br1A	0.086 (3)	0.139 (4)	0.0585 (17)	0.037 (2)	−0.0375 (19)	−0.026 (2)
Br1B	0.0657 (12)	0.087 (2)	0.0686 (17)	0.0096 (11)	−0.0283 (9)	−0.0107 (9)
O7	0.0510 (11)	0.0685 (13)	0.0392 (10)	0.0097 (9)	−0.0088 (8)	−0.0089 (10)
O8	0.0712 (15)	0.104 (2)	0.0630 (14)	0.0406 (14)	−0.0233 (11)	−0.0294 (14)
N1	0.0652 (16)	0.0599 (16)	0.0532 (15)	−0.0119 (13)	−0.0010 (13)	−0.0088 (13)
C13	0.0490 (15)	0.0539 (17)	0.0486 (15)	0.0026 (12)	−0.0027 (13)	−0.0045 (14)
N3	0.0569 (15)	0.0748 (19)	0.0550 (16)	0.0002 (14)	0.0021 (12)	0.0124 (14)
O6	0.124 (2)	0.0891 (19)	0.0434 (13)	0.0033 (16)	0.0012 (14)	0.0082 (13)
C6	0.0503 (15)	0.0517 (16)	0.0384 (14)	0.0037 (12)	0.0008 (11)	0.0024 (13)
O1	0.0849 (17)	0.095 (2)	0.0896 (19)	−0.0353 (15)	−0.0253 (15)	0.0026 (16)
C8	0.0465 (14)	0.0545 (17)	0.0392 (14)	0.0024 (12)	−0.0029 (12)	−0.0010 (13)
C5	0.0476 (15)	0.0572 (17)	0.0470 (16)	−0.0027 (12)	−0.0004 (12)	−0.0035 (14)
O2	0.120 (2)	0.112 (2)	0.0748 (18)	−0.060 (2)	−0.0083 (17)	0.0232 (17)
O4	0.0877 (18)	0.113 (2)	0.0782 (18)	−0.0375 (17)	−0.0326 (14)	0.0049 (16)
O5	0.0850 (18)	0.102 (2)	0.103 (2)	−0.0365 (16)	−0.0261 (16)	0.0474 (18)
C12	0.0588 (17)	0.0632 (19)	0.0469 (16)	0.0008 (14)	−0.0062 (13)	−0.0123 (15)
C7	0.0522 (16)	0.0636 (19)	0.0378 (15)	0.0066 (14)	−0.0042 (12)	−0.0073 (14)
C3	0.0576 (16)	0.0526 (17)	0.0361 (14)	0.0007 (13)	−0.0002 (12)	−0.0006 (13)
C4	0.0478 (15)	0.0589 (18)	0.0392 (15)	0.0009 (13)	−0.0061 (12)	−0.0030 (14)
N2	0.0621 (16)	0.080 (2)	0.0495 (15)	−0.0071 (15)	−0.0141 (13)	0.0007 (15)
C1	0.0439 (14)	0.0572 (17)	0.0326 (13)	0.0046 (12)	−0.0036 (11)	−0.0070 (12)
O3	0.105 (2)	0.112 (2)	0.0749 (18)	−0.0181 (18)	−0.0409 (17)	0.0316 (18)
C2	0.0508 (15)	0.0494 (16)	0.0394 (14)	−0.0040 (12)	0.0011 (12)	−0.0071 (13)
C9	0.0556 (17)	0.075 (2)	0.0479 (17)	0.0163 (16)	−0.0087 (14)	−0.0156 (16)
C10	0.0628 (19)	0.086 (3)	0.058 (2)	0.0252 (18)	−0.0121 (16)	−0.0195 (19)
C11	0.0549 (17)	0.075 (2)	0.0489 (17)	0.0057 (15)	−0.0149 (14)	−0.0107 (16)

### Geometric parameters (Å, º)

**Table:**

Br1A—C11	1.909 (7)	C8—C7	1.469 (4)
Br1B—C11	1.889 (6)	C5—C4	1.371 (4)
O7—C1	1.371 (3)	C5—H5	0.9300
O7—C7	1.379 (4)	O4—N2	1.205 (4)
O8—C7	1.188 (4)	C12—C11	1.380 (5)
N1—O2	1.191 (4)	C12—H12	0.9300
N1—O1	1.205 (3)	C3—C4	1.372 (4)
N1—C2	1.473 (4)	C3—C2	1.376 (4)
C13—C12	1.371 (4)	C3—H3	0.9300
C13—C8	1.389 (4)	C4—N2	1.474 (4)
C13—H13	0.9300	N2—O3	1.217 (4)
N3—O6	1.210 (3)	C1—C2	1.394 (4)
N3—O5	1.222 (4)	C9—C10	1.381 (4)
N3—C6	1.469 (4)	C9—H9	0.9300
C6—C5	1.389 (4)	C10—C11	1.374 (5)
C6—C1	1.392 (4)	C10—H10	0.9300
C8—C9	1.392 (4)		
			
C1—O7—C7	115.5 (2)	C4—C3—H3	120.7
O2—N1—O1	122.8 (3)	C2—C3—H3	120.7
O2—N1—C2	117.4 (3)	C5—C4—C3	122.4 (3)
O1—N1—C2	119.8 (3)	C5—C4—N2	118.8 (3)
C12—C13—C8	120.7 (3)	C3—C4—N2	118.8 (3)
C12—C13—H13	119.6	O4—N2—O3	124.6 (3)
C8—C13—H13	119.6	O4—N2—C4	118.0 (3)
O6—N3—O5	123.5 (3)	O3—N2—C4	117.4 (3)
O6—N3—C6	119.9 (3)	O7—C1—C6	120.8 (2)
O5—N3—C6	116.6 (3)	O7—C1—C2	121.9 (2)
C5—C6—C1	122.1 (3)	C6—C1—C2	117.2 (2)
C5—C6—N3	116.4 (3)	C3—C2—C1	121.9 (3)
C1—C6—N3	121.6 (2)	C3—C2—N1	116.3 (3)
C13—C8—C9	119.7 (3)	C1—C2—N1	121.8 (2)
C13—C8—C7	117.5 (2)	C10—C9—C8	119.5 (3)
C9—C8—C7	122.8 (3)	C10—C9—H9	120.3
C4—C5—C6	117.9 (3)	C8—C9—H9	120.3
C4—C5—H5	121.0	C11—C10—C9	119.6 (3)
C6—C5—H5	121.0	C11—C10—H10	120.2
C13—C12—C11	118.8 (3)	C9—C10—H10	120.2
C13—C12—H12	120.6	C10—C11—C12	121.6 (3)
C11—C12—H12	120.6	C10—C11—Br1B	118.7 (3)
O8—C7—O7	121.0 (3)	C12—C11—Br1B	119.6 (3)
O8—C7—C8	126.3 (3)	C10—C11—Br1A	119.2 (3)
O7—C7—C8	112.7 (2)	C12—C11—Br1A	118.6 (3)
C4—C3—C2	118.6 (3)		
			
O6—N3—C6—C5	161.2 (3)	C7—O7—C1—C2	−83.5 (3)
O5—N3—C6—C5	−21.7 (4)	C5—C6—C1—O7	175.6 (3)
O6—N3—C6—C1	−19.3 (4)	N3—C6—C1—O7	−3.9 (4)
O5—N3—C6—C1	157.8 (3)	C5—C6—C1—C2	−0.1 (4)
C12—C13—C8—C9	2.1 (5)	N3—C6—C1—C2	−179.6 (3)
C12—C13—C8—C7	−176.2 (3)	C4—C3—C2—C1	1.3 (4)
C1—C6—C5—C4	1.0 (4)	C4—C3—C2—N1	−176.6 (3)
N3—C6—C5—C4	−179.6 (3)	O7—C1—C2—C3	−176.7 (2)
C8—C13—C12—C11	−0.9 (5)	C6—C1—C2—C3	−1.0 (4)
C1—O7—C7—O8	−3.2 (5)	O7—C1—C2—N1	1.2 (4)
C1—O7—C7—C8	178.0 (2)	C6—C1—C2—N1	176.8 (3)
C13—C8—C7—O8	1.5 (5)	O2—N1—C2—C3	1.7 (4)
C9—C8—C7—O8	−176.8 (4)	O1—N1—C2—C3	179.0 (3)
C13—C8—C7—O7	−179.9 (3)	O2—N1—C2—C1	−176.2 (3)
C9—C8—C7—O7	1.8 (4)	O1—N1—C2—C1	1.1 (4)
C6—C5—C4—C3	−0.7 (4)	C13—C8—C9—C10	−1.6 (5)
C6—C5—C4—N2	180.0 (3)	C7—C8—C9—C10	176.7 (3)
C2—C3—C4—C5	−0.5 (5)	C8—C9—C10—C11	−0.1 (6)
C2—C3—C4—N2	178.9 (3)	C9—C10—C11—C12	1.3 (6)
C5—C4—N2—O4	8.8 (5)	C9—C10—C11—Br1B	177.4 (4)
C3—C4—N2—O4	−170.6 (3)	C9—C10—C11—Br1A	−169.6 (9)
C5—C4—N2—O3	−172.5 (3)	C13—C12—C11—C10	−0.8 (6)
C3—C4—N2—O3	8.1 (5)	C13—C12—C11—Br1B	−176.9 (3)
C7—O7—C1—C6	101.0 (3)	C13—C12—C11—Br1A	170.2 (8)

### Hydrogen-bond geometry (Å, º)

**Table:**

*D*—H···*A*	*D*—H	H···*A*	*D*···*A*	*D*—H···*A*
C10—H10···O2^i^	0.93	2.48	3.169 (4)	131
C12—H12···O1^ii^	0.93	2.49	3.273 (4)	142
C5—H5···O8^iii^	0.93	2.46	3.384 (4)	173
C3—H3···O6^iv^	0.93	2.42	3.291 (4)	157

Symmetry codes: (i) −*x*+1, *y*+1/2, −*z*+3/2; (ii) −*x*+1, −*y*, −*z*+1; (iii) −*x*+2, *y*+1/2, −*z*+3/2; (iv) *x*, −*y*+1/2, *z*+1/2.
